# Toll Like receptor 7 regulates viral loads and cytokine secretion during acute retroviral infection

**DOI:** 10.1186/1742-4690-9-S2-P174

**Published:** 2012-09-13

**Authors:** E Browne

**Affiliations:** 1 Massachusetts Institute of Technology, Cambridge, MA, USA

## Background

Acute HIV infection is characterized by a high viremia accompanied by a powerful wave of pro-inflammatory cytokines that affects the subsequent course of infection and pathogenesis. Thus, understanding the mechanisms that regulate cytokine secretion and viremia is a key priority. The innate immune receptor TLR7 has been identified a retrovirus-sensing protein, and is expressed in several key immune lineages. In vitro data suggests that HIV can trigger TLR7-dependent innate immune responses, but TLR7s role in vivo is unclear.

## Methods

To determine whether TLR7 affects viremia or cytokine secretion during acute retroviral infection, we analyzed the plasma of wild type and TLR7 deficient mice infected with the model retrovirus, Friend virus (FV).

## Results

We identified 16 cytokines that are significantly upregulated in the plasma of wild-type mice infected with FV, the majority of which are also upregulated during HIV infection. Individual cytokines have distinct kinetic profiles and intensities, with peak levels ranging from 5dpi to 14dpi. To examine the contribution of TLR7 to this response, we compared viral loads and cytokine levels of wild-type or TLR7 deficient mice. Surprisingly, we found that majority of the pro-inflammatory cytokines exhibited exacerbated secretion in the absence of TLR7, while only an early wave of the anti-inflammatory cytokine IL-10 was attenuated. This exacerbated cytokine storm was accompanied by an elevated viremia. Significantly, IL-10 deficient mice also exhibited elevated viremia and cytokine secretion during acute infection. By contrast, TLR7deficient mice exhibit an attenuated antibody response, while anti-viral antibody levels in IL-10 deficient mice were normal.

## Conclusion

Our results demonstrate that TLR7 negatively regulates viral loads and cytokine secretion during acute retroviral infection by promoting an early wave of IL-10, and that TLR7 regulates the development of anti-viral antibodies independently of IL-10. These results reveal that TLR7 plays multiple roles in regulating the immune response to retroviral infection.

